# Time-of-flight camera achieves high diagnostic accuracy in adolescent idiopathic scoliosis: a promising radiation-free alternative to radiography

**DOI:** 10.3389/fbioe.2025.1629872

**Published:** 2025-08-29

**Authors:** André Boché, Anne Tabard-Fougère, Ludmilla Bazin, Mathieu Severyns, Romain Dayer, Tanguy Vendeuvre

**Affiliations:** ^1^ CHU de Poitiers, Service d’Orthopédie-Traumatologie, Poitiers, France; ^2^ Pediatric Orthopedics Unit, Pediatric Surgery Service, Geneva University Hospitals and University of Geneva, Geneva, Switzerland; ^3^ Department of Orthopedics Surgery and Traumatology, Clinique Porte Océane, Les Sables d’Olonne, France; ^4^ Université de Poitiers, CNRS, ISAE-ENSMA, Institut Pprime, Poitiers, France

**Keywords:** time-of-flight camera, adolescent idiopathic scoliosis, trunk asymmetry, validation study, gibbosity

## Abstract

**Background:**

The close monitoring of the adolescent idiopathic scoliosis (AIS) population during the growing years is necessary and requires repetitive X-rays. This study aimed to evaluate the validity and test characteristics of the time-of-flight (TOF) camera, a novel radiation-free tool, for assessing trunk asymmetry in patients with AIS.

**Methods:**

In this prospective diagnostic accuracy study, 94 AIS patients (10–18 years) underwent standardized TOF camera imaging (seated position). Among them, 81 also received an angle of trunk rotation (ATR) assessment using a scoliometer (forward-bending position). The average major Cobb angle (CA) in the cohort was 21.4°. The global trunk asymmetry (GTA) provided by the TOF camera and the scoliometer-based ATR were compared with the gold-standard major Cobb angle from 2D radiography (standing position) using Pearson correlation. Receiver operating characteristic (ROC) analysis evaluated the diagnostic accuracy of GTA for AIS diagnosis (CA > 10°) and brace indication (CA > 20°). Sensitivity (Se), specificity (Sp), and area under the curve (AUC) values were reported and compared for GTA and ATR.

**Results:**

GTA was significantly correlated with the radiographic CA (p < 0.001). For scoliosis diagnosis (CA ≥ 10°), the AUC was 0.87 (0.79–0.96) for GTA (threshold = 7°, Se = 80%, and Sp = 80%) and 0.95 (0.9–1.0) for ATR. The AUC values of GTA and ATR were not significantly different (*p* = 0.129), but sensitivity was significantly lower for GTA (76%) than for ATR (91%). For brace indication (CA ≥ 20°), the AUC was 0.92 (0.86–0.97) for GTA (threshold = 7.87, Se = 93%, and Sp = 76%).

**Conclusion:**

The TOF camera appears to offer promising test characteristics for AIS diagnosis, with a high correlation to radiographic CA and competitive diagnostic accuracy using a scoliometer. Although the seated positioning differs from standard radiographs, this approach enhances reproducibility and patient compliance. The high sensitivity and specificity of the TOF camera for scoliosis diagnosis highlight its potential as a safe, fast, and reliable alternative to X-ray imaging in routine clinical settings. Further investigations (assessing solid screening characteristics and inter- and intra-individual repeatability and validity) are needed before it can replace repetitive radiographs for monitoring AIS progression in growing patients.

## 1 Introduction

Scoliosis is a three-dimensional (3D) spinal deformity combining lateral deviation and axial rotation of the spine (Perdriolle and Vidal, 1987). The definition of scoliosis is based on a Cobb angle (CA) > 10° (James, 1954) measured on a two-dimensional (2D) anteroposterior full-length spine radiograph (X-ray) (Raso et al., 1998).

The overall prevalence of the pathology ranges from 1% to 3% in the population aged from 10 to 18 years (Konieczny et al., 2013). Specific symptoms such as an abnormal appearance, back pain, or psychological problems can be influenced by the cause or the severity of scoliosis and its progression. Most patients with scoliosis are treated with non-surgical therapies, but close monitoring during the growing years is necessary to make sure that no surgery may be required. The use of braces during orthopedic treatment requires X-rays every 4 or 6 months, as recommended by international guidelines (El-Hawary and Chukwunyerenwa, 2014). Repeated radiation exposure by X-rays during this period, coupled with the heightened sensitivity of children to radiation (Kleinerman, 2006), may increase the risk of cancer (Don, 2004).

Reduction in the radiation doses during adolescent idiopathic scoliosis (AIS) surveillance is, therefore, of major importance as a means of avoiding long-term health problems. The recent development of the low-dose 2D X-ray EOS system directly addresses this challenge as it allows a 50%–85% reduction in radiation (McKenna et al., 2012) and has been validated for AIS follow-up (Somoskeöy et al., 2012).

In addition, other radiation-free methods such as 3D ultrasound (Lai et al., 2021), surface topography (Liu et al., 2013), and rasterstereography (Krott et al., 2020) have been explored and validated over the past years for monitoring the evolution of AIS. These techniques offer non-invasive alternatives for evaluating trunk asymmetry and spinal curvature, with varying degrees of correlation to radiographic measurements. However, they often present practical limitations; ultrasound is highly operator-dependent and may lack reproducibility in routine settings, and surface topography and rasterstereography typically require laser scanners or light projections, large physical setups, prolonged acquisition times, and substantial post-processing, which can limit their integration into everyday clinical workflows. More recent systems based on markerless depth-sensing cameras have shown promise but remain constrained by hardware limitations and positioning variability.

In this context, the time-of-flight (TOF) camera evaluated in this study provides a novel, fast, and low-cost solution. It is a range-imaging camera system measuring distances based on time-of-flight, the round-trip time of an artificial light signal, as provided by a light-emitting-diode (LED). It is a compact device without mechanical moving parts, measuring the distance between the camera and the object for each pixel of the image with a single shot. It enables trunk asymmetry assessment in a seated position, with minimal patient cooperation. These advantages would make it a promising tool for routine and radiation-free monitoring of AIS patients in clinical practice.

TOF cameras have been used for several years in many industries such as automotive, aeronautics, and robotics, but their use in the medical field is still under development. The use of a TOF-camera was studied in 2016 for patient positioning during radiotherapy (Gilles et al., 2016) and in 2019 for strict and reproducible electrode localization during an electroencephalogram (Chen et al., 2019). In addition, in 2017, Sharp et al. used it to measure thoracic expansion during pulmonary function tests (Sharp et al., 2017).

To our knowledge, only one other study has used a similar TOF camera-based method to analyze spinal deformity in scoliosis—the study by Mazza et al. (2024), which employed the Spine 3D system (Sensor Medica, Guidonia Montecelio, Italy) based on LiDAR/TOF camera technology. The commonly used screening test is based on the measurement of the angle of trunk rotation (ATR) using a scoliometer during Adam’s forward-bending test (FBT) (Grivas et al., 2007). We, therefore, applied both the TOF camera and the scoliometer to each patient, aiming to compare their measurements as a secondary but valuable objective.

## 2 Methods

### 2.1 Participants

Adolescents were recruited for this study from patients scheduled for biplanar radiography either for suspected scoliosis or for monitoring previously diagnosed scoliosis.

The inclusion criteria were 1) age between 10 and 18 years and 2) informed consent obtained from the participant and their respective legal representatives for photographs. The exclusion criterion was a history of spinal or thoracic surgery.

### 2.2 Radiographic assessment

**FIGURE 1:**
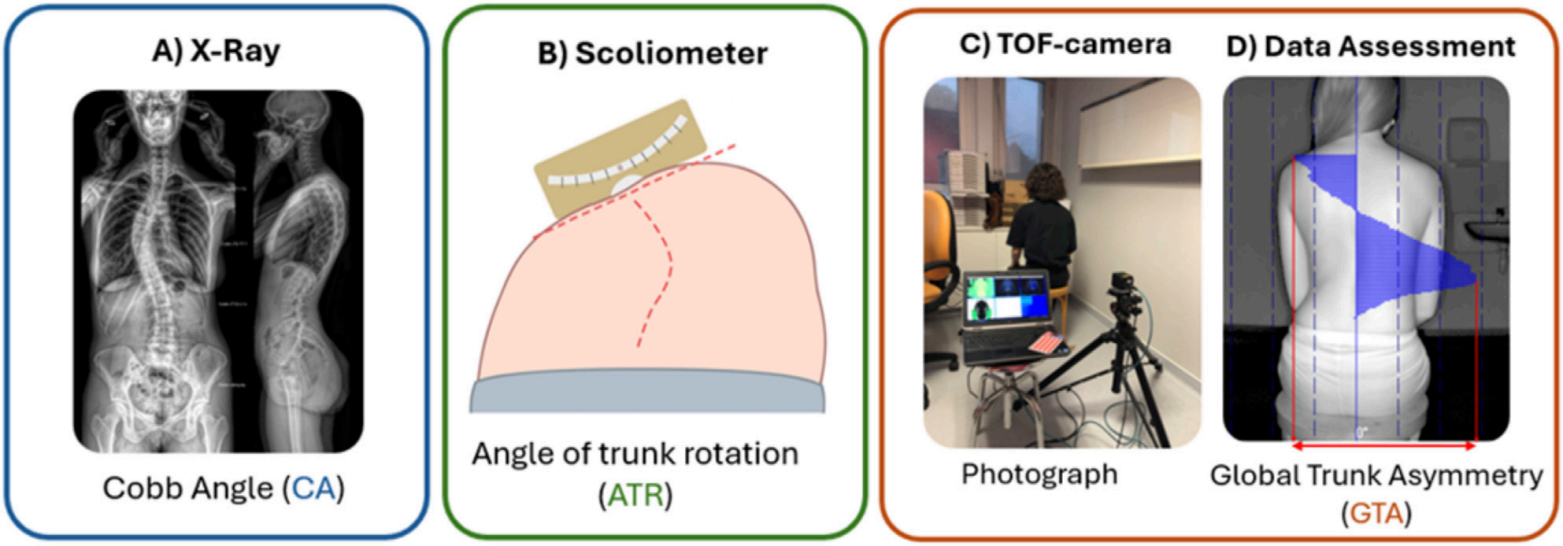
Schematic representation of parameters of interest. **(A)** Postero-anterior biplanar X-ray with the EOS^®^ system (Biospace Med, Paris, France). **(B)** Scoliometer during Adam’s forward-bending test. **(C)** TOF camera setup. The subject is not a patient and is wearing clothes for privacy concerns. **(D)** Blind measurement of GTA using the TOF camera software application.

Each patient had a 2D anteroposterior full-length spine low-dose biplanar X-ray with the EOS^®^ System (Biospace Med, Paris, France) as part of their routine clinical visit. Patients were examined in a standing position with their fists on their clavicles, elbows flexed, and their heads looking forward (Faro et al., 2004) (Figure 1A).

### 2.3 Scoliometer assessment

Adam’s forward-bending test was performed during the clinical visit. The patient had to take off his/her shirt and bend forward, starting at the waist until the back reached a horizontal plane, with their feet together, arms hanging, and knees in extension. The spine surgeon stood behind the patient and looked along the horizontal plane of the spine, looking for the maximal ATR using a scoliometer (Mizuho Co., Ltd., CA, United States), according to the usual guidelines (Fairbank, 2004) (Figure 1B).

### 2.4 TOF camera assessment

The device included the TOF camera, a camera stand, a portable computer, and a stool. All these elements are relatively small and easy to transport, ensuring their mobility and usability in any small room.

The patient was seated on a stool with their back facing the camera (Figure 1C). Ground markers were previously used for stool-positioning reproducibility. The patient had to remove the shirt and bra, if needed, lower the pants and underwear to reveal the lumbosacral region, and put long hair to the front side. The patients were asked to straighten their backs as much as possible and keep their feet parallel to each other, arms flexed with palms on their thighs, and their eyelines straight. Given the exploratory nature of this study, a comfortable and reproducible seated position was intentionally chosen over the standing or Adam’s position, as further discussed. After confirming the patient’s proper positioning on software, the photograph was taken using the computer’s space key. This procedure usually did not exceed 2 min. All the data were saved anonymously in a dedicated folder (Figure 1D).

### 2.5 Data assessment

**FIGURE 2:**
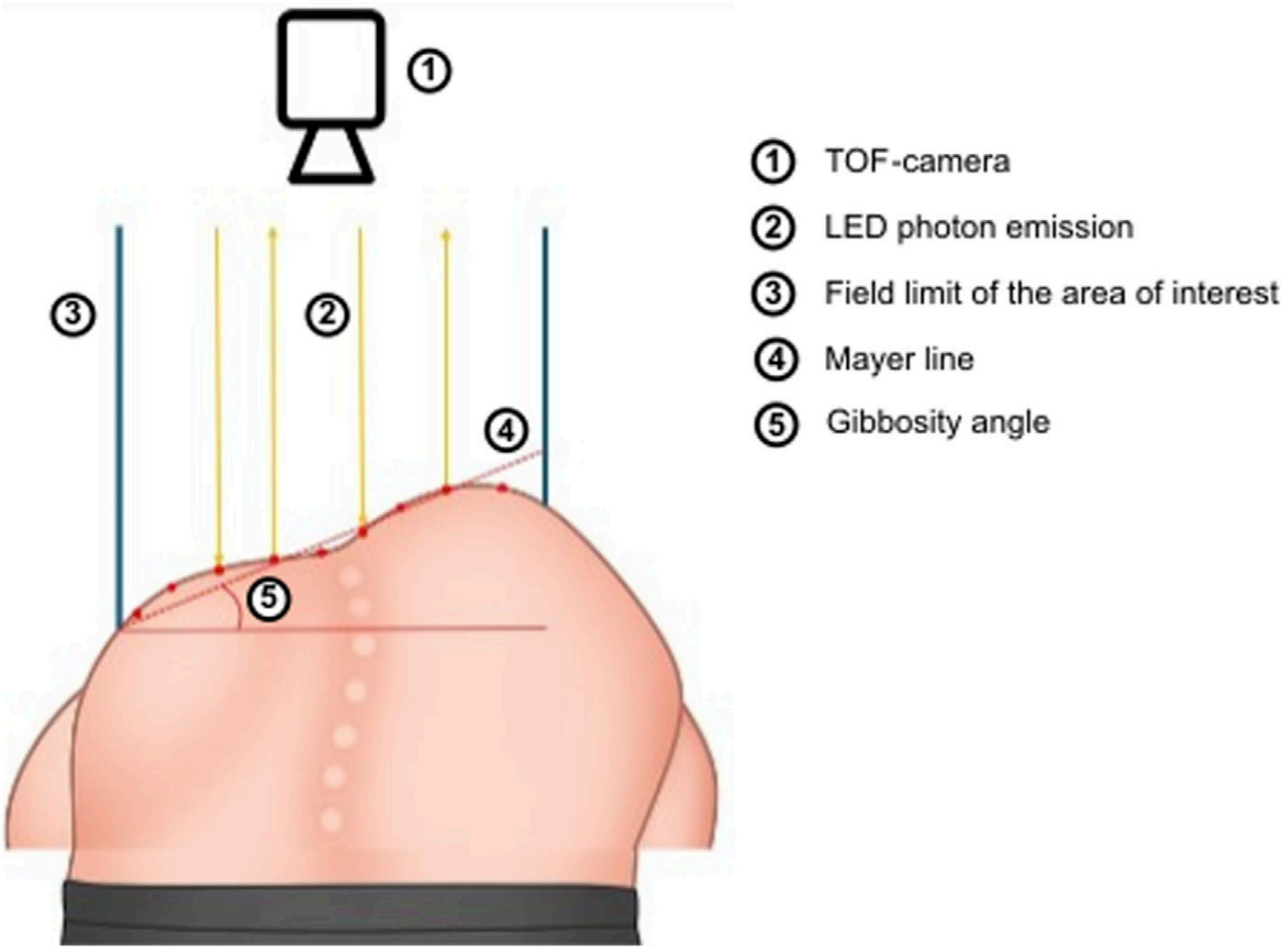
Schematic overview of the TOF camera usage. To improve clarity, the diagram depicts a top–down camera view of a forward-bending patient. In the present study, the camera was placed behind a seated patient, projecting horizontally. This positioning choice is justified in the manuscript.

**FIGURE 3:**
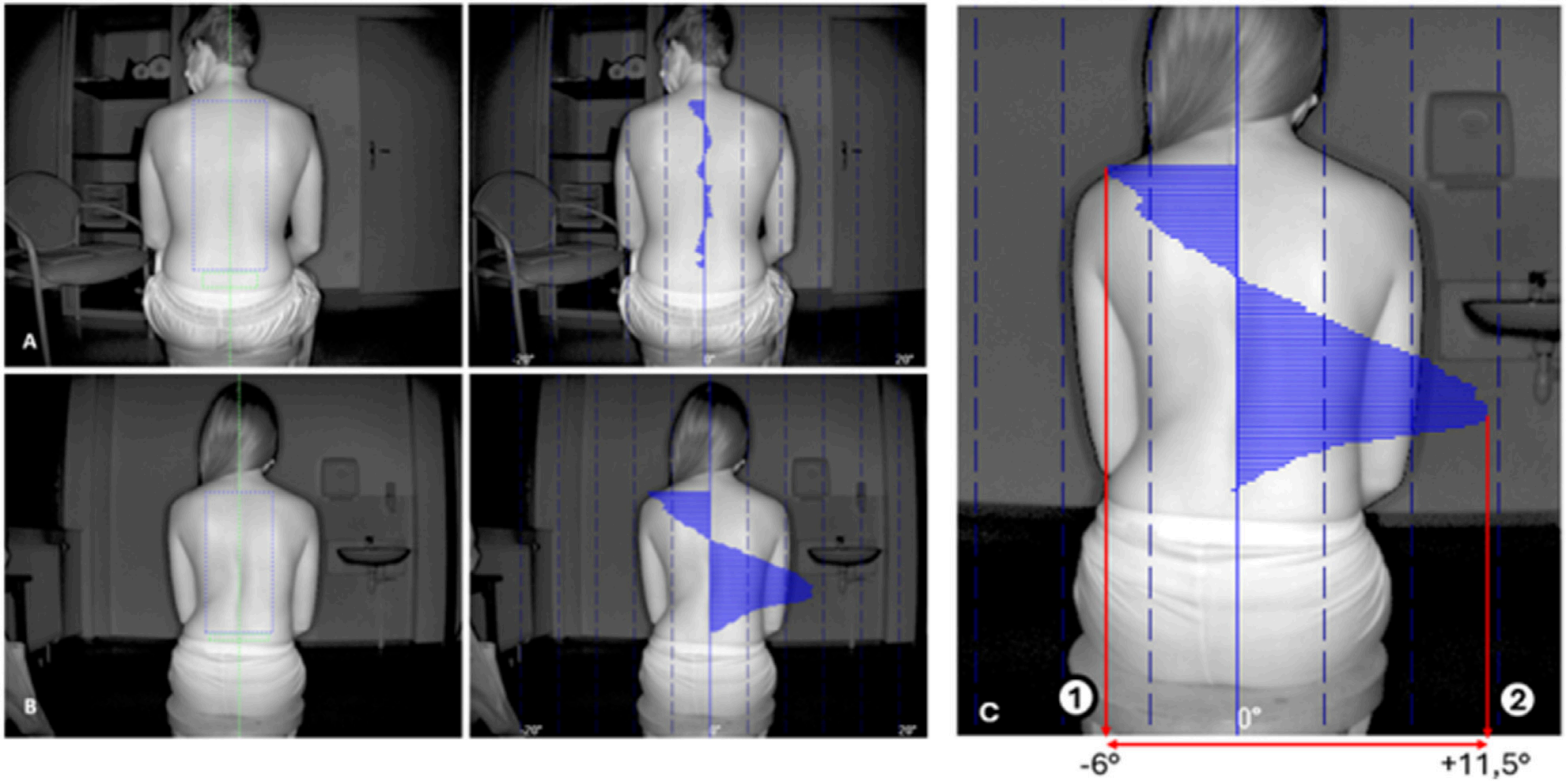
Examples of photograph opened in the software application. The green axis is the symmetry axis, the green rectangle is the reference zone, and the blue rectangle is the area of interest (AI). Seventeen-year-old non-scoliotic patient **(A)**. Twenty-four-year-old patient with right thoraco-lumbarscoliosis (CA > 35°) **(B)**. Gibbosity angles along the height of the AI a re represented by a blue graph. Note the thoraco-lumbar right gibbosity. GTA is the sum of the absolute values of the maximal positive (2) and negative gibbosity (1) angles **(C)**. GTA = 17.5°. Itr represents the overall trunk asymmetry.

TOF camera software estimates an ATR-equivalent angle for each horizontal pixel line within the defined area of interest (AI). This process involved manual steps performed by a blinded operator (Figures 2, 3).

• Symmetry axis: aligned along the spine using the lumbosacral region as a stable reference, and minimally affected by mediolateral deviations.
• Reference zone (RZ): drawn on a flat, lower lumbar/sacrococcygeal area to allow software to re-center the image if the patient’s posture is slightly misaligned.
• Area of interest (AI): defined as high as possible between the scapulae, within a 20-cm band centered on the spine (10 cm on each side), to exclude void spaces and scapular prominences that may distort measurements.

Software outputs gibbosity angles along the vertical axis as a curve. From these, we defined global trunk asymmetry (GTA) as the sum of the absolute values of the maximal positive and negative angles—representing the overall trunk asymmetry. GTA corresponds to the peak angle in single curves and the combined peaks in double curves (Figure 3C).

### 2.6 Primary outcomes

The Cobb angle was blindly measured by a senior spine surgeon on full standing postero-anterior 2D radiographs. The diagnosis was based on a Cobb angle greater than 10° (2), which is considered the gold standard. Parameters selected for the analysis were the GTA provided by the TOF camera and ATR measured using the scoliometer during FBT.

### 2.7 Statistical analysis

All statistical analyses were conducted using R software (version 4.2.2) and related packages (R Core Team, 2024). The continuous outcomes were reported as the mean (standard deviation), and the categorical outcomes were reported as n (%). GTA and ATR measurements were used as primary outcomes. Intra-rater reliability was evaluated for a subgroup of 25 randomly selected patients using intra-class correlation coefficients (ICC, values > 90% indicate excellent reliability) based on a single rater (A.B), absolute-agreement, and a two-way random-effects model. To evaluate the validity of GTA and ATR against the gold standard (X-ray), Pearson correlation coefficients were calculated, along with *p*-values and 95% confidence intervals. The interpretation criteria for correlation were defined as follows: r > 80% was strong, r between 60% and 80% was moderate, r between 30% and 60% was fair, and r < 30% was poor (Akoglu, 2018).

The area under the curve (AUC) statistic from the receiver operating characteristic (ROC) curves was used to describe the discriminative ability of the GTA and ATR parameters for diagnosing AIS (objective #1) defined with a Cobb angle of ≥ 10° (X-ray) and of the GTA for brace indication (objective #2) defined with a Cobb angle of ≥ 20° (X-ray). The ROC curve was generated using the pROC package, including AUC calculation with a 95% confidence interval (Robin et al., 2011). Sensitivity and specificity were reported for the best threshold identified using the Youden method (Youden, 1950). The optimal cut-off point was determined by maximizing the number of correctly classified individuals. The AUCs of GTA and ATR were compared using a bootstrap test for two correlated ROC curve methods, with the roc.test function in the pROC package (Robin et al., 2011). For the GTA, we reported the sensitivity and specificity with the best thresholds. Additionally, we reported values for 7° and 8°.

### 2.8 Sample size estimation

The required sample size was calculated using the ROC curve methodology for diagnostic accuracy studies (pROC package) with Zhou’s prevalence adjustment, assuming an AUC value of 0.80 (versus null AUC = 0.50), 80% power, and α = 0.05 (two-tailed). An anticipated unequal distribution of scoliosis cases was estimated at 80% for objective #1 (CA > 10°) and 50% for objective #2 (CA > 20°: 50%). Based on these parameters, a minimum of 56 participants (45 cases with CA ≥ 10°, 11 controls) were required for objective #1, and a minimum of 100 participants (50 cases with CA ≥ 20° and 50 with CA < 20°) were required for objective #2. Our final enrollment of 94 participants exceeded the number needed for objective #1 (achieving 98.2% power for observed AUC = 0.87) while providing 87% power for objective #2 (compensated by a higher observed AUC = 0.92).

## 3 Results

### 3.1 Participants

Patients ranged in age from 10 to 18 years (mean 14.2 years) and were predominantly women: 67% (63 F and 31 M) (Table 1). The average body mass index (BMI) was 18.9, ranging from 14.8 to 28.5. Among the 94 evaluated patients, 79 (87%) had scoliosis based on X-ray-measured CA ≥ 10°. The others did not have scoliosis and were considered our control population. Without bending X-rays, we were unable to sort the patients by the Lenke curve types (Lenke et al., 2001). We considered four curve types, but only for descriptive purposes. A total of 23 patients had a thoracic main curve, 17 had a lumbar main curve, 14 had a double major curve, and 22 had a thoracolumbar main curve.

**TABLE 1:** Population description.

Variable	Total (n = 94)
Female, n (%)	63 (67%)
Mean age, years	14.2 (1.6)
Risser sign >1, n (%)	73 (78%)
Mean BMI (range)	18.9 (14.8–28.5)
Mean major Cobb angle, degrees	21.4 (14.7)
Curve type	
Main thoracic (MT), n (%)	23 (24%)
Main thoracolumbar (TL), n (%)	22 (24%)
Main lumbar (ML), n (%)	17 (18%)
Double major curve (DM), n (%)	14 (15%)
Double thoracic (DT), n (%)	3 (3%)
No scoliosis curve (Cobb < 10), n (%)	15 (16%)
Mean ATR, degrees	7.1 (4.1)
Mean GTA, degrees	8.9 (4.2)

The results are reported as the mean (standard deviation) for the continuous outcomes and n (%) for the categorical outcomes. GTA is the global trunk asymmetry evaluated using a time-of-flight camera, and ATR is the maximal angle of trunk rotation evaluated using a scoliometer.

According to the 2012 epidemiologic study by Konieczny et al. (2013), thoracic curves are the most common (48%), followed by thoracolumbar/lumbar curves (40%). Double curves (9%) and double thoracic curves (3%) are less common, while 80% of all children have thoracic or thoracolumbar/lumbar curves (4). Our population does not appear to differ substantially from the overall idiopathic scoliosis population.

Ninety-four patients were analyzed using TOF camera software. A total of 13 out of the 94 patients had missing data concerning the ATR measured with the scoliometer; 81 patients were consequently compared for the best threshold. The intra-rater reliability for the GTA was excellent (ICC = 0.97% [95% CI: 94%–99%], with a standard measurement error of 0.76.

### 3.2 Comparison with the major Cobb angle (X-ray)

**FIGURE 4:**
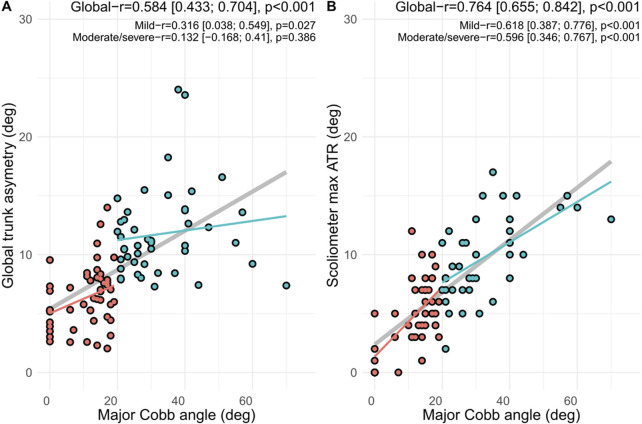
Correlation between radiographic evaluation of the major Cobb angle (gold standard) and **(A)** global trunk asymmetry using the TOF camera and **(B)** maximal angle of trunk rotation (ATR) using a scoliometer. r is the Pearson correlation coefficient; mild curves are defined with a Cobb angle of < 20°, and moderate/severe curves are defined with a Cobb angle of ≥ 20°.

As shown in Figure 4, the Pearson correlation coefficients for GTA and max ATR were 0.584 and 0.764, respectively, suggesting moderate correlation with the major Cobb angle (X-ray). Considering the mild curves (Cobb < 20°), a fair correlation (r = 0.316, *p* = 0.03) was reported for GTA and a moderate correlation (r = 0.618, *p* < 0.001) was reported for ATR compared to the major Cobb angle (X-ray).

### 3.3 Diagnostic characteristics

**FIGURE 5:**
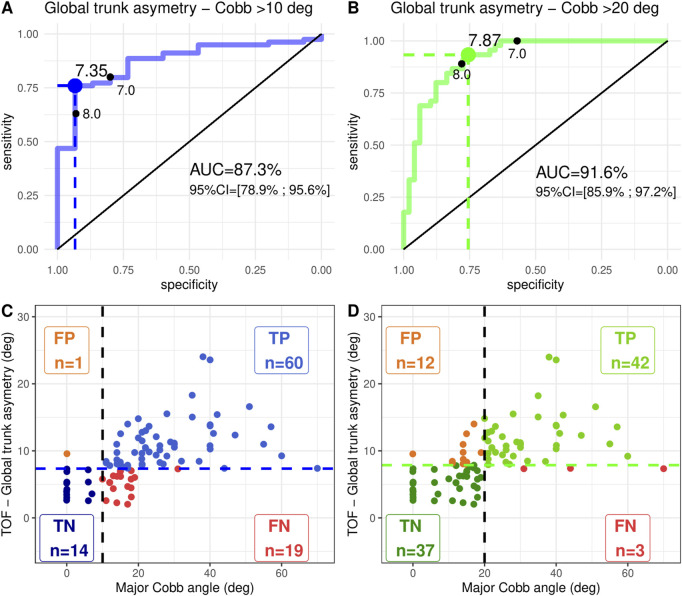
First line illustrates the AUC statistic from ROC curves with the associated best cut-off point (Youden method) of the global trunk asymmetry using the TOF camera. The second line describes the test characteristics of the best cut-off point for **(A-C)** idiopathic scoliosis diagnosis defined with a Cobb angle of ≥ 10° (X-ray) and **(B-D)** brace indication defined with a Cobb angle of ≥ 20° (X-ray). Deg is degrees; 95% CI is the corresponding 95% confidence interval; FP is false positive; TP is true positive; TN is true negative; FN is false negative*.*

**FIGURE 6:**
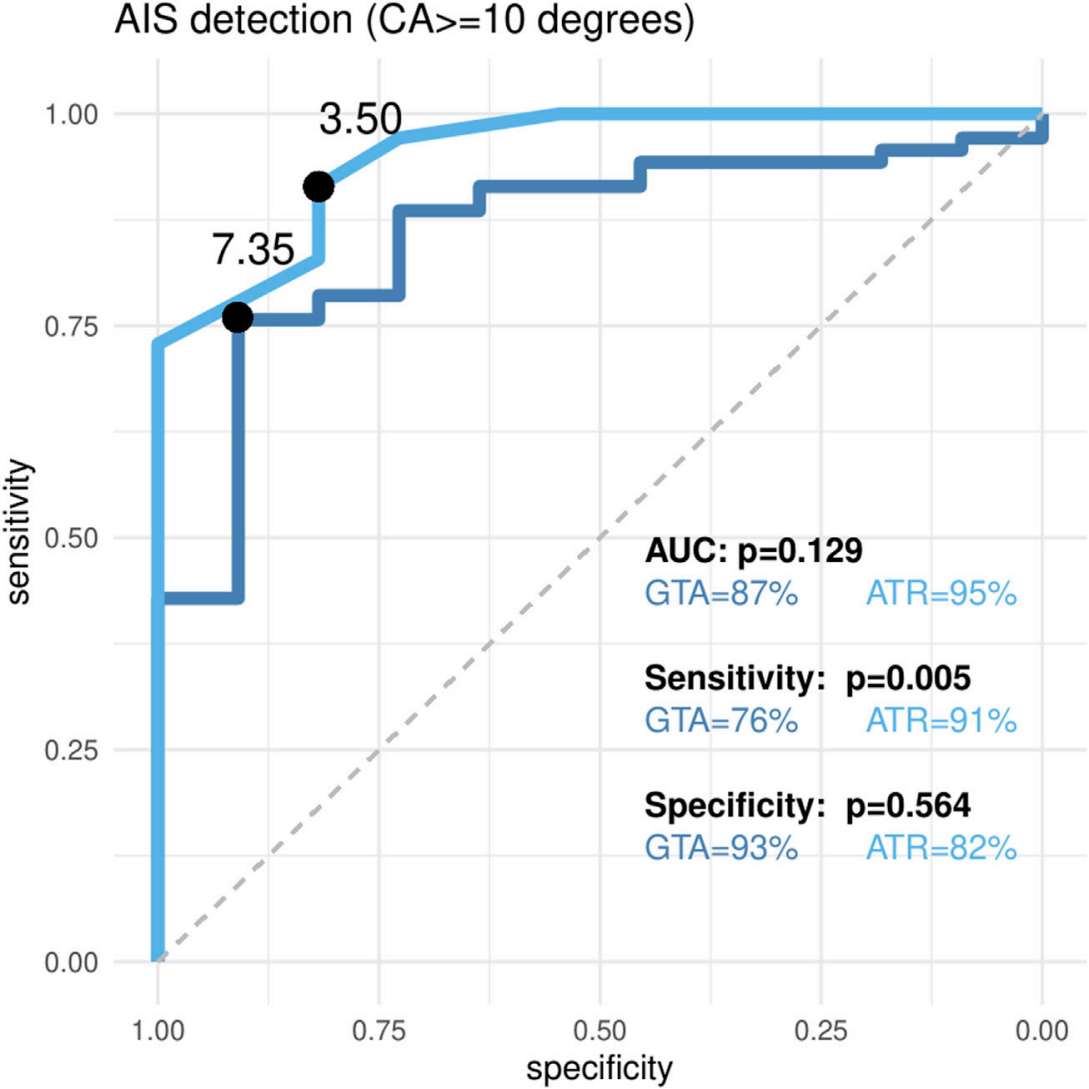
AUC statistic from ROC curve comparison between global trunk asymmetry using the TOF camera and the maximal angle of trunk rotation (ATR) using a scoliometer for idiopathic scoliosis diagnosis defined with a Cobb angle of ≥ 10° (X-ray). Significant differences between AUC were considered at *p* < 0.05. AUC is reported with the respective 95% confidence interval*.*

As summarized in Figures 5, 6, considering scoliosis diagnosis (CA ≥ 10°), the AUC value was 0.87 (0.79–0.96) for GTA and 0.95 (0.92–1.0) for ATR. Considering CA ≥ 20° as the cut-off for orthopedic treatment, the AUC value was 0.92 (95% CI = 0.86–0.97) for GTA.

Considering the diagnosis of CA ≥ 10°, the sensitivity and specificity of GTA for a threshold of 7.35 were 76% (65%–85%) and 93% (68%–100%), respectively (Table 2). The sensitivity and specificity of GTA for the threshold <7 were 80% (69%; 88%) and 80% (52%; 96%), respectively. For the threshold <8, they were 63% (52%; 74%) and 93% (68%; 100%), respectively.

**TABLE 2:** Diagnostic accuracy results of the GTA evaluated with a TOF camera to classify curves as idiopathic scoliosis (Cobb angle ≥ 10°) and idiopathic scoliosis with brace indication (Cobb angle ≥ 20°) for 94 patients.

Variable	Scoliosis diagnosis Radiograph		Brace indication Radiograph	
	Cobb ≥ 10° (+)	Cobb < 10° (−)	Cobb ≥ 20° (+)	Cobb < 20° (−)
GTA<7	Se = **80%** (69%; 88%); Sp = **80%** (52%; 96%)		Se = 100% (92%; 100%); Sp = 57% (42%; 71%)	
+	63	3	45	21
-	16	12	0	28
Best cut-off	Se = 76% (65%; 85%); Sp = 93% (68%; 100%)		Se = **93%** (82%; 99%); Sp = **76%** (61%; 87%)	
+	60	1	42	12
-	19	14	3	37
GTA<8	Se = 63% (52%; 74%); Sp = 93% (68%; 100%)		Se = 89% (76%; 96%); Sp = 78% (63%; 88%)	
+	50	1	40	11
-	29	14	5	38

The best cut-off point was identified using the Youden method on the ROC curve: 7.35 for scoliosis diagnosis (Cobb angle ≥ 10°) and 7.87 for brace indication (Cobb angle ≥ 20°). Se is sensitivity, and Sp is specificity reported with 95% confidence intervals.

Considering CA ≥ 20° as the cut-off point for orthopedic treatment, the sensitivity and specificity of GTA for a threshold of 7.87 were 93% (82%–99%) and 76% (61%–87%), respectively. The sensitivity and specificity of GTA for the threshold <7 were 100% (92%; 100%) and 57% (42%; 71%), respectively. For the threshold <8, they were 89% (76%; 96%) and 78% (63%; 88%), respectively.

The AUCs of GTA and ATR were not significantly different (*p* = 0.129) (Figure 6). Using the Youden method, the optimal threshold for GTA was 7.35, yielding a sensitivity of 76% (65%–85%), which was significantly lower than that of ATR (threshold = 3.5, sensitivity = 91% [82%–97%]; *p* = 0.005). Specificity, however, did not differ significantly between the two methods, with both values exceeding 80%.

## 4 Discussion

This study evaluated GTA test characteristics with a TOF camera for the diagnosis of idiopathic scoliosis in 94 patients compared with the gold standard (the major Cobb angle measured with 2D-radiography). Considering the diagnosis of scoliosis (CA ≥ 10°) and brace indication (CA ≥ 20°), the TOF camera achieved clinically acceptable accuracy (AUC > 85%, sensitivity/specificity > 80%) with thresholds of 7.35 and 7.87, respectively, with the potential to reduce radiographic monitoring.

The correlation between GTA and the major Cobb angle was studied as a prerequisite for subsequent analyses. The global Pearson coefficient was 0.584, which can be considered a moderate correlation; although the value was higher for ATR, it was still considered moderate. If a patient is classified as non-scoliotic with GTA under the threshold, an X-ray may not be obtained, possibly leading to the repercussions of insufficient treatment. The number of false negatives is a major issue and may be due to the high number of CA between 9 and 11, with patients who could easily be misclassified between the ill and not-ill categories. The trunk deformation of two patients with a 1° or 2° discrepancy may be too low to be detected by the TOF camera. This discrepancy may also be due to operator errors when measuring CA on X-rays. The same drawback arises when we consider diagnostic accuracy. GTA with a threshold of 7 appears to offer better screening characteristics (sensitivity 80% and specificity 80%) than with the Youden best threshold 7.35 (sensitivity 76% and specificity 93%), given that the sensitivity is superior for the scoliosis diagnosis. Considering CA > 20 as a brace indication cut-off point, the Youden threshold of 7.87 appears to be preferable since specificity decreases to 57% for a threshold of 7. Thresholds 7, 7.35, 7.87, and even 8 are very close to one another, and significant differences in diagnostic accuracy appear for narrow differences of GTA values. Concerning the diagnosis of scoliosis (CA ≥ 10°), the significantly higher sensitivity of ATR suggests that it may be preferable in clinical contexts where minimizing false negatives is critical.

In a recent study using the same methodology, rasterstereography, with a scoliosis angle threshold of 12.5°, offers interesting screening characteristics for AIS (Vendeuvre et al., 2023). In this study, the sensitivity of rasterstereography was 75% with a specificity of 65%. Among these two radiation-free methods, the TOF camera appears to be a better alternative.

In the TOF camera process, the operator must ensure that the patient follows all the required positioning rules and that the capture button can be pressed by any medical or non-medical member of the screening team. However, to obtain the GTA, the operator must manually define three zones using software. In our study, only one operator performed this step blindly. The same applied to Cobb angle measurements on X-rays, which could interfere with diagnostic accuracy, especially when GTA values are close. Although the pilot assessment of intra-operator consistency supports measurement stability (ICC > 90%, standard error of measurement <1.0°), a formal reliability analysis would be valuable for future studies. Inter-operator repeatability of GTA evaluation with the TOF camera remains to be explored in further studies.

To respect patient privacy, the sacrococcygeal region is often covered by clothing and cannot be fully exposed. It may affect the operator’s choice of the interest zone or reference zone.

The strength of the present study resides in the inclusion of 94 patients, 50 aged between 10 and 14 years and 44 aged between 15 and 17 years. They represent the population targeted in school-based screening tests (Riseborough and Wynne Davies, 1973) and the population with scoliosis, which requires regular follow-up consults. This study also included patients without scoliosis (15 out of 94, 16%) who underwent X-rays. This allowed us to calculate the intrinsic characteristics of the test for the early diagnosis of scoliosis. Most of the literature includes few or no healthy control patients to define them (Fong et al., 2010). Recruiting additional healthy controls would have required unnecessary exposure to ionizing radiation, which raised ethical concerns and limited further inclusion.

Another strength of this study lies in the comparison with the most widely used clinical tool, the scoliometer, even though the study was not originally designed for this comparison. The AUCs of GTA and ATR were very good (0.87 and 0.95) for scoliosis diagnosis and do not significantly differ (*p* = 0.129) (Figure 6). This is a promising result for the TOF camera since the diagnostic characteristics of the scoliometer in our study appear to match those of the literature (Côté et al., 1998). This comparison is nonetheless mainly informative. The TOF camera evaluates the back surface of a seated patient in a more natural position, allowing us to negate the effect of leg-length inequality, but at the detriment of gibbosity. The ATR of the scoliometer is measured with the patient leaning forward, exacerbating gibbosity. For a more relevant technical comparison, the photograph would need to be taken with the TOF camera pointing vertically toward the ground during FBT. However, we believe this may undermine the reproducibility of the measurements. It might also render it more difficult to establish a reference zone, such as the sacrococcygeal region. While both GTA (TOF camera) and ATR (scoliometer) showed strong diagnostic performance, we believe that the TOF camera offers a faster, operator-independent, and potentially more consistent measurement of trunk asymmetry.

Our study shares a common technological foundation with the work of Mazza et al. (2024), who employed a LiDAR-based system (Spine 3D^®^) for surface topography analysis in scoliosis. Although both studies rely on comparable surface imaging technology, they differ significantly in terms of methodological design. Our study included a larger cohort, with the addition of a control group, allowing for broader clinical relevance and comparative analysis. We also incorporated radiographic validation through Cobb angle measurements, strengthening the reliability of our results.

Compared to systems such as rasterstereography or 3D ultrasound, which often require complex setups, trained operators, or time-consuming processing, the TOF camera offers a fast, low-cost, and user-friendly alternative. Its acquisition is simple and reproducible and requires minimal patient preparation. Although the current analysis of GTA remains semi-automated, the pipeline could be designed to support full automation. Future developments will aim to enable a fully integrated “one-click” solution, from image acquisition to clinical output, making the tool even more feasible for routine AIS monitoring in clinical practice.

Several limitations in this study require consideration. One of our main concerns is the comparison between 2D standing radiographs and the TOF camera measurements taken in a sitting position. Since this is the first pilot study using the TOF camera for scoliosis assessment, we aimed to explore the feasibility and potential of this technology. The sitting position was chosen to establish a preliminary understanding of the system’s diagnostic performance. It minimizes patient movement and ensures a more consistent positioning, leading to improved reproducibility of the measurements. It is also more comfortable for young patients, especially those with significant spinal deformities, making the imaging process less stressful and more patient-friendly. Finally, the sitting position also neutralizes the impact of any limb length discrepancy, which can affect the measurement of spinal alignment in standing radiographs. This allows for a more accurate assessment of trunk asymmetry without the confounding influence of lower-limb variations (Kotwicki et al., 2007). We fully acknowledge that future studies should explore the use of the TOF camera in a standing position for better comparability with standard radiographs.

The other main limitation of this study is the fact that participants were included from a tertiary referral center, with patients referred by pediatricians, general practitioners, and school doctors or nurses for suspected AIS. This does not represent the general population and limits the generalizability to population-wide screening. Predictive values are important diagnostic characteristics, and due to this drawback, they were not evaluated.

Two patients had BMI values of 27 and 28.5, which, although technically below the adult obesity threshold of 30, are classified as obese based on age-adjusted WHO criteria. No subgroup analysis was performed for these cases due to insufficient statistical power. However, Margalit et al. (2017) recently found better sensitivity of the scoliometer for an ATR of 7° in normal patients than for 5° in obese patients. This suggests that high BMI would tend to hide curvatures, justifying the referral for specialist consultation with lower ATR. Future studies evaluating the screening performance of the TOF-based systems should account for BMI as a potential confounding factor.

Before concluding, we must keep in mind that patients and their families are more concerned with improving the external shape or esthetics than with their Cobb angle. For esthetic issues, the shoulder, scapula, waist, or breasts should be considered (Zaina et al., 2009), and their asymmetry would probably be better evaluated with a surface topography technique, such as the TOF camera, than via traditional radiological parameters.

## 5 Conclusion

The TOF camera appears to offer interesting test characteristics (sensitivity and specificity > 80%) for scoliosis diagnosis with a 7° threshold. Due to their non-radiation and non-invasive characteristics, surface topography techniques have great potential in assessing scoliosis. Among these techniques, we believe that the TOF camera can play an important role. It is an inexpensive, lightweight, compact, and easily transportable device. It does not require any major installation, and it allows easy and fast image acquisition, making it interesting for routine use. Further investigations are needed (assessing solid screening characteristics, inter- and intra-individual repeatability, and the ability to detect curve progression over time) so that the TOF camera can be used instead of repetitive radiographs for monitoring the evolution of AIS in growing patients.

## Data Availability

The raw data supporting the conclusions of this article will be made available by the authors, without undue reservation.
